# Optimizing the Droplet-Vitrification Procedure by Balancing the Cryoprotection and Cytotoxicity of Alternative Plant Vitrification Solutions Based on the Nature of Donor Plant Vigor

**DOI:** 10.3390/plants12234040

**Published:** 2023-11-30

**Authors:** Haenghoon Kim

**Affiliations:** Department of Agricultural Life Science, Sunchon National University, Suncheon 57922, Republic of Korea; cryohkim@scnu.ac.kr; Tel.: +82-061-7505185; Fax: +82-061-7503210

**Keywords:** A3-80%, B5-85%, cryopreservation, liquid overlay, shoot tip, systematic approach, three-step regrowth, two-step preculture

## Abstract

Over 30 years of plant vitrification, droplet vitrification (DV) of in vitro propagules and slow freezing of dormant buds are typical methods of large-scale cryobanking worldwide. One-step sucrose preculture and Plant Vitrification Solution 2 (PVS2) cryoprotection in solution-based vitrification often face unacceptably low regeneration, and the results are on a case-by-case basis depending on the plant species, like a blind test. The absence of a universal protocol applicable across all plant diversity is considered one of the limiting factors. For wild flora, limits of source material available and difficulties in in vitro propagation make it worse to re-optimize the protocol steps for new species. Since cryoprotectant toxicity is the most crucial barrier to the vitrification of organized explants, selecting alternative plant vitrification solutions (PVS) based on the cytotoxicity of cryoprotectants is vital. This review proposes the concept of donor plant vigor (DPV), which refers to the donor plant properties that determine the potential to regenerate normal plantlets under various cryopreservation procedures. DV is a multi-stage procedure with many factors from stage (1) material preparation to (2) pre-liquid nitrogen (pre-LN) (preculture, osmoprotection, cryoprotection), (3) LN (cooling), (4) warming conditions (rewarming, unloading), and (5) regrowth. Since the cytotoxicity of PVS is a primary limiting factor in DV approaches, DPV is crucial for coping with the toxicity of PVS. The DPV is innate and can be maximized with appropriate material preparations, i.e., vigorously growing in subcultures aided by a liquid overlay on top of the gelled medium, selecting proper explants, optimizing the two-step preculture conditions, and media supplements. Developing the DV protocol starts with testing the material with a tentative standard protocol, which includes a two-step preculture (10% sucrose for 31 h and 17.5% sucrose for 16 h), osmoprotection with C4-35%, cryoprotection with A3-80% (60 min at 0 °C), cooling, and rewarming using aluminum foil strips. Using a three-step regrowth initially with ammonium-free regrowth medium, regrowth of shoot tips in one plate following the successive stages of the tentative standard protocol for shoot tips, i.e., fresh, PC, OP, CP (LNC), and LN, is a valuable tool to characterize the sensitivity of the material and to standardize the procedure by tuning the cryoprotection and cytotoxicity of cryoprotectants. A-series PVS (A3-90%, A3-80%, A3-70%) and B-series PVS (PVS3, B5-85%) can be tested based on the DPV. These alternative PVSs have been applied in over 30 pieces of literature with an 8.5~67.3% increase in LN regeneration compared to PVS2 and Plant Vitrification Solution 3 (PVS3) treatments. Using this approach as an alternative to blind condition screening would be influential in broadening the cryopreservation of diverse wild species and problem materials.

## 1. Introduction

Cryopreservation, i.e., the storage of viable material in liquid nitrogen (LN, −196 °C) offers a valuable option for the long-term conservation and use of non-orthodox or limitedly available seeds and vegetatively propagated species [1,2]. Since vitrification-based plant cryopreservation was reported in the early 1990s [3,4], studies have expanded to crops, biotechnology products, endangered wild species, and algae [2]. Over 15 thousand accessions have been cryobanked worldwide, mainly using in vitro propagules through a droplet-vitrification (DV) [5] method or using dormant buds [6].

The triangle of cryopreservation is plant material, cryopreservation protocol, and manipulation skills [7]. Regarding the plant material, survival varies greatly amongst species and genotypes [8]. As the cytotoxicity of cryoprotective agents (CPAs) is the main barrier to plant vitrification, the compatibility of the materials to CPA mixtures is critical. Like seed vigor [9], donor plant vigor (DPV) for cryopreservation may refer to the donor plant properties that determine the potential for rapid, uniform regrowth and regeneration of normal plantlets under various cryopreservation procedures, especially cryoprotection with highly concentrated CPA mixtures. Hence, DPV is a determining factor for higher LN regeneration. This review introduces a strategy to increase the DPV through the vigorous growth of donor plants during subculture by applying a liquid overlay onto the Gelrite-gelled medium and in a two-step preculture and practically balancing the cryoprotection and cytotoxicity of CPA mixtures, which resulted in a soft landing of regrowth from untreated control (fresh), preculture (PC), osmoprotection (OP), cryoprotection (CP) with Plant Vitrification Solution (PVS, LNC),cooling, and rewarming (C/W, LN).

Most cryopreservation studies have focused on developing protocols using a limited number of genotypes (personal observation). As a solution-based vitrification method, DV is a multi-stage procedure with many factors from stage (1) material preparation to (2) pre-LN (preculture, osmoprotection, cryoprotection with vitrification solution), (3) LN (cooling), (4) warming (rewarming, unloading), and (5) regrowth. However, the mainstream of the literature focuses on the optimization of stage 2 {preculture (sucrose concentration, duration), cryoprotection (PVS2 duration)} (personal observation). Cytotoxicity to Plant Vitrification Solution 2 (PVS2) depends entirely on the species, and even genotype; thus, the optimum incubation time depends upon the plant species, explant conditions, temperature, and preculture conditions. Theoretically, the optimum CPA concentration depends on the explants’ sensitivity to the cytotoxicity of CPAs, while the incubation time is flexible to the size or permeability of the explants. Therefore, with medium-sized shoot tips, comparing different components and concentrations of CPA mixtures is worth investigating. In this regard, choices of alternative PVSs, which will reviewed in this review, broaden the applicability to plant kingdoms.

If these approaches are not satisfiable, researchers carry out multiple-condition screening or try to test other methods, such as vitrification [3], encapsulation–dehydration (ED) [4], encapsulation–vitrification (EV) [10], and cryo-plates [11,12], which re related to stage 3 (cooling and warming containers) [13]. Since DV of in vitro propagules is a science and art, the operational manipulation skills at stages 1–5 are one determining factor that guarantees high, stable post-LN regeneration. Becoming a master or trying less sensitive protocols, like cryo-plates, can be a feasible solution. V cryo-plates [11] and D cryo-plates [12] are a hybrid of aluminum foil strips (AFS, DV) and encapsulation (EV or ED) that have the advantages of rapid cooling and rewarming (C/W) using tiny beads and less sensitivity for non-skillful hands, respectively.

The regrowth of cryopreserved explants (Stage 5) has gained attention recently [14], with an emphasis on reactive oxygen species (ROS)-induced oxidative stress, antioxidants, and initial ammonium-free regrowth medium. During the vitrification procedure, explants are susceptible to cytotoxicity (chemical toxicity, osmotic stress), freezing injury, etc., and these injuries may trigger oxidative stress, stress-related gene expression, and reduced metabolic rate, which peaks at LN (stages 3 and 4). Thus, ammonium ions in the regrowth medium are initially toxic for 5–7 days, and improved regrowth has been established by eliminating ammonium in the first 5–7 days [15].

This review proposes a systematic approach to design directions for a standard protocol for new plant materials using alternative PVSs of different concentrations of CPA mixtures based on the DPV.

## 2. Vitrification and Plant Vitrification Solutions (PVSs)

### 2.1. Solution-Based Vitrification

Cryopreservation can be classified into slow cooling (inter-cellular freezing) and rapid cooling (vitrification). Vitrification solidifies a liquid into a noncrystalline or amorphous solid (glass) [16], like a snapshot of the liquid state [17]. Explants must be exposed to highly concentrated cryoprotectant mixtures (vitrification solution, VS) and high-speed C/W for the vitrification.

While incubating with highly concentrated VS, explants efflux the water (dehydration) and influx the CPAs (loading) to escape the ice crystallization and recrystallization. During the C/W, explants traverse the danger zone (−15~−60 °C), where ice crystallization and recrystallization may occur (0 to −40 °C) [18]. Solutions often crystallize during rewarming, called devitrification, even though the ice crystal was suppressed during freezing [19]. This accounts for metastable vitrification due to the cytotoxicity of VS, which means that the explants are insufficiently dehydrated for vitrification. Hence, rapid cooling and, more critically, rewarming rates are needed to avoid injuries [20]. The critical warming rates increase exponentially as the CPA concentration decreases [21].

In this regard, using AFS as a C/W container (DV) proved a better option than using cryovials (vitrification), which are associated with very rapid rewarming of foil strips to pre-heated (40 °C) unloading solution as a prerequisite for higher regeneration [18,22,23]. The surface-area-to-volume ratio can be increased since a drop of VS covers AFS-mounted explants. Accordingly, heat transfer can be enhanced, like ultra-rapid cooling (low-CPA vitrification) [24]. Low-CPA vitrification requires a high concentration of CPA (4–8 M or 65% *w/v*) with a cooling rate of 10^−3^~10^−6^ °C min^−1^ [24]. In DV, cooling rates using AFS range from 100 to 500 °C s^−1^ [18]. The necessity of ultra-rapid C/W implies that the explants are insufficiently cryoprotected by VS primarily due to the cytotoxicity, which limits the CPA concentration and exposure time. In combination with rewarming directly in a pre-heated (40 °C) unloading solution, AFS facilitates the rewarming rate and produces the highest post-LN regrowth [22]. Seki and Mazur also suggested that a high warming rate is more critical than a cooling rate [25].

### 2.2. CPAs Action and Toxicity

According to the permeability of the cells, CPAs are classified into permeating and non-permeating types [26,27]. Permeating CPAs, such as dimethyl sulfoxide (DMSO), ethylene glycol (EG), and glycerol (relatively slower), can cross cell walls and plasma membranes and thus may induce intrinsic biochemical toxicity. They reduce ice formation and growth and cause cell dehydration [26]. Permeating CPAs penetrate through the cell wall and induce temporary plasmolysis, and when they pierce the protoplasm, the cells plasmolyze [26,28].

Though sucrose is classified as a non-permeating CPA in animal models, including polyethylene glycol (PEG) and soluble proteins [27,29], it is reasonable to consider semi-permeating CPAs in plant systems [30]. Semi-permeating CPAs, such as sucrose and trehalose, may penetrate the cell walls but not the plasma membranes and, thus, induce osmotic damage [26]. Sugars regulate the shape and delay the ice crystal growth, in addition to ice-recrystallization inhibition properties [29]. Although trehalose has been more frequently used as a non-permeating CPA than sucrose in animal models [31], sucrose was more effective in ice blocking (inhibiting the nucleation and growth of the crystals) in the model vitrification solution of 60% glycerol than other disaccharides, like trehalose [31]. Sucrose has additional advantages in the plant model since it has an excellent affinity for plants as a phloem transporter of photosynthetic assimilates and low-temperature resistance-related metabolites, and thus is relatively less toxic to plant cells [32]. With the aid of the semi-permeating CPA sucrose, a less-poisonous amount of permeating CPAs is needed for vitrification [33].

Cryoprotectant toxicity is the most crucial barrier to the vitreous preservation of complex, spatially extended organisms [20]. Many variables can influence CPA toxicity, including the CPA mixture type, concentration, temperature, and exposure time [34]. On a molal basis, glycerol and EG are the least toxic CPAs, followed by DMSO in singular and two-component CPA mixtures, compared to others, such as propylene glycol, formamide, etc. A three-component CPA mixture of glycerol + DMSO + EG showed the lowest toxicity rate at seven molal concentrations [34]. The cytotoxicity induced by VS can be considered osmotic, biochemical, or both [28]. Osmotic damage results from cell volume change caused by a too-rapid introduction (loading) and removal (unloading) [28], and too-high osmoticum per se can be a biophysical toxicity. The intrinsic biochemical toxicity may result from specific interactions with the biochemical constituents of the cell or from nonspecific effects of biomolecules [28]. The average water hydrogen bonding of the polar groups within the molecular structure (*qv = M*_W_*/M*_PG_, where *M*_W_ is the molarity of water and *M*_PG_ is the molarity of polar groups on permeating CPAs) is considered a parameter of CPA toxicity [20].

### 2.3. Plant Vitrification Solutions

Vitrification solution is the central part of the solution-based vitrification procedure. There is some literature on PVSs, their composition, and their performance on plant materials [27,30,34,35,36]. The two-factor hypothesis suggests that biological samples encounter mechanical damage (intracellular ice formation) and solute damage (solute effects or dehydration caused by chemical and osmotic effects of concentrated solutes in the residual unfrozen water between ice crystals) in cryopreservation [27,37]. Though the vitrification procedure can avoid ice crystallization during the cooling process with the aid of highly concentrated CPAs in the PVS, the ice recrystallization or devitrification occurring during the rewarming stage may cause fatal damage to the cryopreserved samples [20]. It is challenging since the cytotoxicity induced by the concentrated PVS may not permit sufficient cryoprotection with the PVS in organized plant tissues and organs, like shoot tips and bulblets [17]. Hence, the main barrier is the cytotoxicity induced by the PVS [28,38]. Therefore, the choice of PVS, which has a strong vitrification tendency with less cytotoxicity, whatever osmotic shock and biochemical toxicity, is crucial in plant cryopreservation.

During cryoprotection, the highly concentrated CPA solution (PVS) effluxes the water and dehydrates the explants from 80–90% to 30–50% while in-fluxing permeating CPAs into the explant [36,37]. In the DV procedure, the moisture contents of fresh garlic shoot apices were decreased from 84% to 38% with PVS3 for 150 min at room temperature (RT), and at the same time, permeated CPA glycerol and sucrose in PVS3 reached 175 mg g^−1^ FW and 128 mg g^−1^ FW, respectively [39]. The concentration of glycerol and sucrose influx was highly negatively correlated with the moisture content of the cryoprotected garlic apices (*r* = −0.99 in both cases) [39]. These CPAs may replace some water molecules inside the cell [33]. With the permeation of CPAs, *Rubia akane* hairy roots initially shrink (efflux of water) and swell again (influx of CPAs) and restore osmotic equilibrium [27,40].

Sucrose preculture improved the tolerance to both cryoprotection (LNC) and cryopreservation (LN), possibly by acquiring dehydration and freezing tolerance [41]. The osmoprotection treatment may (biophysically) mitigate the osmotic shock occurring in the plant samples through exposure to concentrated PVSs [42,43]. Considering their composition and concentration (Table 1), PVS2-based solutions (A-series, four components) evoke primarily chemical toxicity and, additionally, osmotic stress, while PVS3-based solutions (B-series, two components) mainly induce mainly osmotic pressure [44,45].

Due to the cytotoxicity, explants are not sufficiently cryoprotected and thus subject to ice crystallization (cooling) and ice recrystallization (rewarming), contributing to cell death and leading to osmotic stress [46]. Ice recrystallization describes the increase in ice crystal size over time in an already-frozen material. Even in the best scenario, vitrification, a glassy and amorphous state without ice nucleation, is metastable and thus prone to devitrification during rewarming. Hence, rapid cooling and, more critically, rapid rewarming are the keys to plant vitrification [21]. Cooling using AFS and rewarming in a pre-heated (40 °C) unloading solution was critical for LN regrowth of potato shoot tips [22].

**Table 1:** Composition of solutions used for preculture, osmoprotection, cryoprotection, and unloading in droplet-vitrification procedure.

Stage	Solution *	Composition (%, *w/v*) **	Total Concentration (%, *w/v*)	References
Preculture	S-10%	S 10.0	10.0	-
	S-17.5%	S 17.5	17.5	-
Osmoprotection	C4-35%	G 17.5 + S 17.5	35.0	[42]
	C6-40%	G 20.0 + S 20.0	40.0	[42]
	C7-32.1%	G 18.4 (2 M) + S 13.7 (0.4 M)	32.1	[42]
	C-Nii-38.9%	G 18.4 (2 M) + S 20.5 (0.6 M)	38.9	[12]
Cryoprotection	A1-73.7% (PVS2)	G 30.0 + DMSO 15.0 + EG 15.0 + S 13.7	73.7	[3]
	A3-90%	G 37.5 + DMSO 15.0 + EG 15.0 + S 22.5	90.0	[44]
	A3-80%	G 33.3 + DMSO 13.3 + EG 13.3 + S 20.1	80.0	[47]
	A3-70%	G 29.2 + DMSO 11.7 + EG 11.7 + S 17.4	70.0	[41]
	A7-90%	G 37.5 + DMSO 10 + EG 10 + S 32.5	90.0	[44]
	B1-100% (PVS3)	G 50.0 + S 50.0	100.0	[43]
	B5-85%	G 42.5 + S 42.5	85.0	[48]
	B5-80%	G 40.0 + S 40.0	80.0	[44]
Unloading	S-35%	S 35.0	35.0	[49]

* Names of the solutions used in the references. ** G, glycerol; S, sucrose; DMSO, dimethyl sulfoxide; EG, ethylene glycol. All solutions were prepared based on the culture medium used; pH was adjusted to 5.8 before filter sterilization.

## 3. Alternative Plant Vitrification Solutions (PVSs) Based on the Nature of Donor Plant Vigor

### 3.1. PVS2 and PVS3 and Their Variants

A four-component PVS2 [3] has been the most frequently used PVS, and the incubation time is notably variable depending on the species, incubation temperature, explant size and permeability, and conditions of the cryopreservation procedure [30,36]. One of the difficulties with some species is that they are susceptible to the cytotoxicity of PVS2; thus, the optimum incubation time is very short (10~25 min), making the manipulation difficult [1,50]. Cryoprotection at 0 °C is less toxic than at RT due to the lower permeability, but some species are sensitive even at 0 °C [30,41,44,50]. In this case, LN regeneration is prone to go down due to cytotoxicity and insufficient cryoprotection. Therefore, a less-concentrated and less-toxic CPA mixture can be a valuable alternative. A two-component PVS3 [43] has also been used for more extensive desiccation-tolerant materials [45].

Kim et al. designed eight variants of PVS2 (A-series) and four of PVS3 (B-series) and applied these alternative PVSs to garlic and chrysanthemum shoot tips [44]. A3-90%, one of the PVS2 variants, has a higher concentration of glycerol (30%→37.5% *w/v*) and sucrose (13.7%→22.5%) compared to the original PVS2 (A1-73.7%), while DMSO and EG remained 15% each. When the three PVSs were 80% diluted from their initial concentration, the endothermic melting enthalpies of 80%-diluted PVS2, A3-90%, and PVS3 were −35.9 vs. −0.3 vs. −3.1 J g^−1^, respectively, which indicated that the thermal properties of A3-90% were superior to those of PVS2 and PVS3 [44]. When garlic apices (3 × 3 mm) were cryoprotected with PVS2, A3-90% (30 min at RT), and PVS3 (150 min at RT), the moisture content decreased from 84% to 67.0% vs. 60.8% vs. 37.6%, respectively. With the same condition, the endothermic enthalpies of garlic apices cryoprotected with PVS2, A3-90%, and PVS3 were −16.7 vs. −8.2 vs. −1.3 J g^−1^ FW, respectively. The LN regeneration cryoprotected with PVS2, A3-90%, and PVS3 was 50.2% vs. 69.9% vs. 98.8%, respectively [44]. With the increased concentration of glycerol and sucrose, the glass-forming tendency of A3-90% solution cryoprotecting garlic and chrysanthemum shoot tips was better than that of PVS2 [44].

### 3.2. Application of Alternative PVSs

These alternative PVSs have been applied in more than 30 pieces of literature with or without direct comparison with PVS2 and PVS3 from 2009 to 2023, as listed in Table 1 and summarized in Table 2. These alternative PVSs have been applied mainly to DV and a few cases of vitrification [47,51,52,53], EV [49,54], and V cryo-plate methods [55,56,57,58]. The usefulness of alternative PVSs was confirmed in encapsulation-based procedures, too. Compared to the non-encapsulated explants (DV), encapsulated *R. akane* hairy roots (EV) need highly concentrated vitrification solutions {A3-70% → A3-90%, B5-80% → B1-100% (PVS3)} and longer durations (20 min at 0 °C → 40–80 min) for the highest LN regeneration [49].

In general, PVS3 is suitable for more extensive and harder explants (bulbs, rhizomes) such as *Allium sativum* [44], *Lilium* spp. [64], and *Cymbidium kanran* [52], and also for materials susceptible to biochemical toxicity, such as *Chrysanthemum morifolium* [44,59] and *Solanum tuberosum* [61]. The sensitive and soft materials, both biochemically and osmotically with smaller sizes, respond well with a diluted PVS3, i.e., B5-80%; for example, hairy roots of *R. akane* [45] and shoot tips of *Aster altaicus* [70] and *Penthorum chinense* [48].

In earlier applications in fruit trees, A3-90% at RT resulted in lower regeneration of cryoprotected control (LNC, 10~50%) and cryopreserved (LN, 10~38.2%) than the PVS3 in *Rubus fruiticisus* [60], *Prunus cerasifera* [60], *Lithodora rosmarinifolia* [62], and *Rubus cerasus* × *P. canescens* [63]. The causes may include primarily severe cytotoxicity of A3-90% at RT and partly inappropriate preculture conditions of lower sucrose concentration or shorter duration.

But when A3-90% was applied at 0 °C, LN regeneration (55~66%) increased and was comparable to PVS3 in *R. fruticosus* [67], *Castilleja levisecta* [68], and *A. altaicus* [70] and superior to PVS2 in *Prunus* sp. (A3-90% 39–56% vs. PVS2 8-17%) [51] and *Malus* sp. (75% vs. 40%) [53]. In biochemically sensitive materials of *C. morifolium* and *S. tuberosum*, cryoprotection with PVS2 at RT is very toxic [44,59,61]. Therefore, a variant A7-90% (lower DMSO and EG, higher glycerol and sucrose) was less harmful and produced higher LN regeneration than PVS2 in *Chrysanthemum* (A7-90% 65.3% vs. PVS2 30.8%) and *Solanum* (80.9% vs. 21.7%) [59,61].

Since A3-90% contains a higher concentration of glycerol and sucrose than PVS2, facilitating the efflux of water (dehydration) and the influx of CPAs (loading), it has a higher glass-forming tendency but drives the plant material into severe cytotoxicity, even when cryoprotected at 0 °C. Later, hence, less-concentrated PVSs A3-80%, A3-70%, and B5-80% (less toxic with mild chemical toxicity and osmotic stress) were applied successfully to shoot tips of the sensitive species *L. rosmarinifolia* [62], *C. morifolium* [69], *A. altaicus* [70], *Fragaria* × *ananassa* [72], *Postemon yatabeanus* [74], *P. chinens* [48], and *Dendrathema grandiflourum* [7].

*P. chinense* shoot tips were susceptible to chemical toxicity and moderately sensitive to osmotic stress. Therefore, a lower-concentration PVS A3-70% (52.3%) produced the most increased LN regeneration compared to PVS2 (41.1%), A3-80% (36.8%), and B5-80% (40.9%) in the first round of studies. But after the rejuvenation of donor plants by inoculation of apical sections and overlay of the liquid medium on top of the Gellan gum-gelled medium (liquid-overlay), another variant of PVS B5-85% resulted in the highest LN regeneration (64.2%), which was higher than the A3-80% (53.6%) and PVS2 (45.5%) [48]. Recently, in chrysanthemum shoot tips, A3-80% (85.4%) showed the highest LN regeneration, followed by, in descending order, B5-85% (78.8%) > PVS2 (66.2%) > A3-90% (56.7%) > PVS3 (43.9%) with their optimized conditions [7]. These comparative studies have enabled pilot cryobanking of the chrysanthemum and sweet potato collections at Sunchon National University and back-up at the National Agrobiodiversity Center, Korea.

A tiny and soft material, *R. akane* hairy roots cryoprotected with less-concentrated PVSs at RT, i.e., B5-80% (20 min, 79.5%) and A3-70% (10 min, 45%), produced significantly higher LN regeneration than the B3-90% (37.1%) > A3-80% (27.5%) > PVS2 (10 min, 21%) > A3-90% (0%) [41]. This result indicates that cytotoxicity is the primary hurdle for this susceptible material. When the roots were cryoprotected with A-series PVSs for 20 min on ice, LN regeneration was not significantly different: A3-70% 83.8%, PVS2 73.3% [76].

With insufficient cryoprotection using diluted PVSs due to their cytotoxicity, using AFS over cryovials is more critical in hairy roots of *R. akane* (86.3% vs. 58.8%) [65], shoot tips of *A. altaicus* (56.1% vs. 4.5%) [70] and *P. yatabeanus* (88% vs. 11%) [74], and shoots of *P. chinense* (35.0% vs. 12.5%) [48] and *D. grandiflourum* (86.7% vs. 55.6%) [7].

In comparison, tiny clumps of *Kalopanax septemlobus* embryogenic callus were tolerant to cryoprotection with both A-series (40 min at 0 °C) and B-series (40 min at RT) PVSs and, thus, all highly concentrated PVSs produced high LN regeneration: A3-90% 99.8%, A3-80% 99.3%, PVS3 95.2%, PVS2 94.6%, B3-90% 92.5%%. In contrast, the less-concentrated PVSs A3-70% (15.4%) and B5-80% (57.8%) resulted in poor responses [47]. The thermal analysis evidenced these LN regeneration data: no enthalpies on cooling and nil (−0.1~−0.9 J g^−1^ FW) on rewarming for the former highly concentrated PVSs; no~−67 J g^−1^ FW on cooling and −13.0~−20.4 J g^−1^ FW on rewarming for the latter. The embryogenic callus was tolerant to cytotoxicity, allowing sufficient cryoprotection with concentrated PVSs of both A- and B-series, and thus no difference in LN regeneration between AFS (DV) and cryovials (vitrification) [47].

The conflicting results between the hairy roots and the embryogenic callus account for the differences in the sensitivity of the materials to the PVSs. The choices of alternative PVSs thus can be based on the innate sensitivity of the material to chemical toxicity and osmotic stress on the one side and the size and permeability of the explants on the other side [76].

Though most plant species’ shoot tips are moderately sensitive, the determinant criteria for whether the material is sensitive or tolerant to biochemical toxicity and osmotic stress are unclear. As Fahy [77] pointed out, the cryoinjury is correlated not with the amount of ice formed but with the concentration of the permeating CPAs; the main hurdle in plant vitrification is the toxicity of the PVS applied which, consequently, can result in insufficient cryoprotection with PVS. Therefore, the principal options to investigate are balancing or tuning the cryoprotection and cytotoxicity. However, the mainstream in plant cryopreservation studies has investigated PVS2 duration. The cytotoxicity of CPA mixtures varies depending on the biochemical characteristics of the CPAs and their interaction with the plant material. Even in the same species and genotype, the toxicity sensitivity may vary according to the growth pattern of the donor plants in subcultures, preconditioning, and explants (position, age). Therefore, researchers may use systematic approaches to select proper PVSs based on the sensitivity of the plant materials to PVSs (DPV) and improve the DPV.

### 3.3. Application of Alternative Osmoprotection Solutions

Kim et al. designed seven osmoprotection solutions (OSs, namely, loading solutions) and compared them with het conventional OS C7-32.1% (2 M glycerol + 0.4 M sucrose) [42]. The melting enthalpies and moisture content of cryoprotected garlic apices were not different between osmoprotected and non-osmoprotected, indicating that the effect of osmoprotectant comes from the mitigation of osmotic shock before cryoprotection with PVS, rather than the inhibition of freezing injury [42,45]. Hence, the concentration of OS may not be significant for osmotically tolerant materials.

However, for susceptible plant material, the duration of PVS incubation should be short, and, in that case, OS treatment may allow CPAs to permeate the inner part of tissues to stabilize the samples biochemically [42]. Therefore, the composition of the OS affected LN regeneration for osmotically sensitive materials. C4-35% (balanced glycerol and sucrose) produced higher LN regeneration than the conventional OS C7-32.1% in hairy roots of *R. akane* (72.4% vs. 51.1%) [41], shoot tips of *C. morifolium* (C4-35% 65.3% vs. C7-32.1% 53.9%) [42], and *Clinopodium odorum* (C4-35% 57.4% vs. C7-32.1% 50.2% vs. C-Nii-38.9% 36.2%) [56].

## 4. Strategies to Cope with Cytotoxicity of PVSs

Since the primary hurdle of cryo-injury is the compatibility of shoot tips to cytotoxicity induced by cryoprotection with highly concentrated PVSs, the key to successful cryopreservation is characterizing the innate DPV and optimizing the DPV during subculture and preconditioning. The DPV is inherent and can be maximized during the donor plant preparation and pre-LN stages. Practical approaches to maximizing the DPV may include growing the donor plants vigorously through the optimization of subcultures, preconditioning of donor plants (cold hardening, pre-growth), selection of proper explants, and precise preculture to cope with the cytotoxicity of PVS treatment.

Rapid growth and, eventually, high dry weight with no or little growth hormones may indicate a higher DPV, since the explants from these donor plantlets can tolerate the cytotoxicity of PVSs, resulting in higher LN regeneration than the lower DPV [78]. Cold hardening of donor plants is often employed to improve cryotolerance, increasing membrane lipids’ unsaturation ratio [79,80].

As discussed previously, the strength of cryoprotection (CPA concentration, duration) is limited to the tolerance level to the cytotoxicity of PVS. Indeed, innately tolerant material can be cryopreserved using full-strength cryoprotectants A3-90% [53] or PVS3 [44], guaranteeing higher LN regeneration. These species can tolerate chemical cytotoxicity and osmotic stress, respectively. But, most species studied showed no less than some osmotic and chemical sensitivity. In this case, there are several approaches to adapting the plant material to these stresses during the procedure, i.e., vigorous growing of donor plants (subcultures), two-step preculture, balancing cryoprotection and cytotoxicity (pre-LN), rapid C/W and proper unloading (LN, warming), and regrowth initially with ammonium-free medium (regrowth) [7,15,36,74,78].

### 4.1. Liquid Overlay-Induced Donor Plant Vigor (DPV)

The importance of healthy donor plants is common sense in the plant cryobiologist community. There is a lot of literature on the vigor of donor plants (subculture conditions and duration, growth), preconditioning of donor plants (cold acclimation, pre-growth, preculture), and explants (type and location, size) [81,82,83,84,85,86]. Engelmann [80] and Reed [87] also pointed out that choosing starting material and cultural conditions is as necessary as the cryopreservation protocol.

Though the preparation of vigorous donor plant material is the starting point for successful cryopreservation, in vitro growth of donor plants is influenced by many factors, such as the source material, medium, growth hormones, culture conditions, age of the culture, etc., and in vitro cultures often grow slim and slowly at initiation and spontaneously during the subcultures. Therefore, establishing the appropriate subculture system is a prerequisite not only for multiplication but also for DPV.

The plantlet cultures may grow poorly for diverse reasons: improper culture systems, delaying of the subculture interval, unskillful manipulations, or even unknown causes. In this case, inoculating the apical section instead of the node and adding a liquid medium on the Gelrite-gelled medium has been applied spontaneously for two decades in the lab to rejuvenate the donor (stock, mother) plantlets. It has been proven to significantly improve the growing velocity and dry weight of subcultured donor plants and subsequent LN regeneration in *P. yatabeanus* [78], *P. chinense* [48], and *D. grandiflourum* [7]. Liquid overlay was used as a standard subculture in *A. altaicus* [70].

**Figure 1:**
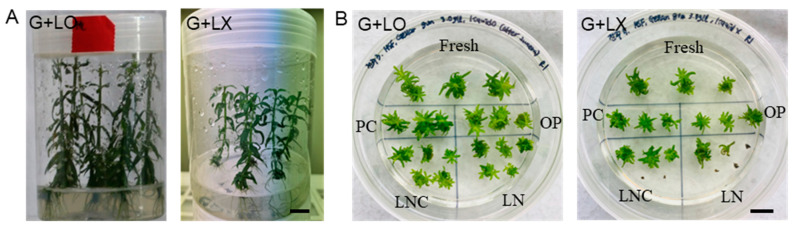
(**A**) In vitro subcultured *Pogostemon yatabeana* plantlets for 5 weeks with Gelrite-gelled medium + liquid overlay (G + LO) and no liquid overlay (G + LX). (**B**) One plate of *P. yatabeana* shoot tips after the standard droplet-vitrification procedure of fresh, PC, OP, LNC, and LN in a regrowth medium of RM1 (ammonium-free MS) for 5 days and RM2 for 3 weeks plus 2 days. Subculture conditions: G+LO, Gelrite 3.0 g L^−1^ + liquid overlay; G + LX, Gelrite 3.0 g L^−1^ + no liquid. Fresh, fresh control shoot tips; PC, preculture with 10% sucrose for 31 h; OP, PC and osmoprotected with C4–35% for 40 min; LNC, PC-OP and cryoprotected (CP) with A3-80% for 60 min at 0 °C, but not cryopreserved; LN, PC-OP-CP and cryopreserved (LN). Scale bar 10 mm [78].

In vitro growth (height, dry weight) of *P. yatabeana* plantlets was affected by diverse factors of the subculture medium and culture conditions (overlay of liquid medium, gelling agent, medium strength, growth hormones, activated charcoal, and subculture duration) [78]. Moreover, the subculture conditions critically determined LN regeneration (18.3~89.7%); the more vigorous the growth, the higher the LN regeneration. The dry weight of donor plants’ shoots and roots and subsequent LN regeneration were highly correlated (*r* = 0.85–0.98) among the treatments tested [78]. The shoot tips subcultured with a liquid overlay on the gelled medium improved the growth of the donor plants (height 3.43 cm → 7.0 cm, dry weight 1.3 mg → 4.8 mg) (Figure 1A(G + LO)) and subsequently, caused higher LN regeneration of node-induced shoot tips (35.8% → 89.7%) (Figure 1B(G + LO)) than the no-liquid overlay (Figure 1A(G + LX),B(G + LX)) [78]. It is worth noting that the beneficial effect of liquid overlay is a little bit of vigorous growth of shoot tips from the fresh, PC, and OP and eventually higher LNC (cryoprotected control) and LN. The higher LN regeneration of heavy-dry-weight donor plantlets accounts for the tolerance to cryoprotection with VS A3-80%, rather than the reduced freezing injuries. Cytotoxicity has been considered a bottleneck in the vitrification-based cryopreservation of complex spatial explants [17]. It has been recognized that small, actively dividing juvenile tissue with dense cytoplasm and less vacuolized cells is prone to tolerate freezing injury [85,88].

Even with the liquid overlay subculture, the shoot tips of *P. chinense* plantlets were sensitive to the cytotoxicity of the PVS. Thus, a lower level of cryoprotection (A3-70% for 30 min at 0 °C) was the optimum condition of cryoprotection, with an LN regeneration of 49.8%. After two years of in vitro maintenance, another personnel rejuvenated the plantlets with two cycles of subculture, i.e., inoculating the apical sections instead of the nodal sections and liquid overlay onto the gelled medium (apical section + liquid overlay). Rapidly growing rejuvenated plantlets shortened the subculture duration from 7 weeks to 5–6 weeks. Node cutting-induced shoot tips tolerated a higher level of cryoprotection (A3-80% 60 min at 0 °C), and B5-85% for 60 min RT produced the most increased LN regeneration (64.2%) among the cryoprotection conditions, which implies the shoot tips of rejuvenated donor plants became more tolerant to osmotic stress [48].

In cryobanking of chrysanthemum (*D. grandiflourum*), though the average LN regeneration of 154 accessions was 74.8%, four accessions showed relatively lower LN regeneration with noticeably weak and slow growth during the subcultures. The 1–2 cycles of apical section + liquid overlay revitalized the DPV and increased the LN regeneration from 44.4–50% to 75.0–76.9% [7].

Liquid overlay on a gelled medium, in other words, double-phase culture, displays the advantages of the solid and liquid medium while eliminating both systems’ disadvantages in pear, ginger, and pine trees [89,90,91,92]. The liquid overlay may replenish beneficial substances and dilute inhibitory ones [91,93]. However, recent studies imply that a liquid overlay may modulate the osmotic potential of the gelled medium and in vitro plantlets and increase the dry weight by facilitating mixotrophic metabolism, i.e., primarily heterotrophic plus slightly autotrophic. Direct contact of the stems with a liquid medium (water, sucrose, minerals, and other substances) works, too [78]. Not only the full-strength liquid medium supplemented with MS nutrients and sucrose but also distilled water (neither MS nutrients nor sucrose) significantly increased the dry weight and eventually gave higher LN regeneration (unpublished data). Liquid overlay likely provides beneficial effects, especially in the case of poorly established in vitro root systems. Further investigations are expected, especially on the extended applications and their mechanism of liquid overlay.

### 4.2. Two-Step Preculture

One of the principal approaches to increasing plant materials’ compatibility with cytotoxicity is sucrose preculture; mostly 10% (0.3 M) sucrose for one or a few days (one-step) has been dominantly applied [54,56,66]. Sucrose preculture may compensate for the effect of cold acclimation [94]. The osmolarity of 10% sucrose (0.420 Osm) is slightly higher than that of the in vitro plant tissue supernatant, which induces mild osmosis. During preculture, plant cells experience osmotic stress, ultrastructural changes, stress-related metabolites, and proteomic responses [95,96,97].

Though 10% sucrose is a good option for initiating osmotic adaptation, an additional higher sucrose concentration can be better for desiccation adaptation if the explants can tolerate this higher concentration. Therefore, a two-step preculture, 10% sucrose for one day followed by 17.5% (0.5 M) sucrose overnight, is recommended for testing. Depending on the explants’ response, 10% sucrose can be extended up to a few days, but no more than the sprouting.

The two-step preculture produced 10–23% higher LN regeneration than one-step preculture with 10% sucrose (Table 2): *C. morifolium* [S-10% (30 h) → S-17.5% (16 h), 38.8% vs. S-10% (30 h), 18.7%, 68], *C. levisecta* [S-10% (17 h) → S-17.5% (4 h), 82.5% vs. S-10% (24 h), 59.5%, 67], *A. altaicus* [S-10% (55 h) → S-17.5% (17 h), 69.2% vs. S-10% (48h), 47%, 61], *D. grandiflourum* [S-10% (31 h) → S-17.5% (16 h), 86.7% vs. S-10% (48 h), 76.3%, 7]. One exception is that *P. yatabeana* shoot tips are sensitive to S-17.5%, and thus, S-10% (88%) produced higher LN regeneration than S-10% → S-17.5% (65%) or no preculture (57%) [78].

Since the impact of sucrose preculture and PVS is connected, the beneficial effect of alternative PVS A3-90% and A3-80% over PVS2 was remarkable when the shoot tips were two-step precultured. Indeed, two-step preculture may increase the usefulness of PVS3 and its modification, B5-85%, which induces dominant osmotic stress. Certainly, these B-series PVSs are a valuable option for materials susceptible to chemical toxicity but less susceptible to osmotic stress. A higher concentration, 25% (0.7 M) sucrose, can be tested on solid, extensive explants, such as bulbils and rhizomes, and rarely on osmotically tolerant shoot tips.

### 4.3. Three-Step Regrowth Initially with Ammonium-Free Medium

For the unloading of CPAs, explants were washed once or step-wise using unloading solutions. Step-wise medium change daily from 10.3~20.5% sucrose to standard (3% or even lower) sucrose medium was applied for various cell cultures and some shoot tips to facilitate the removal of CPAs and toxic chemicals [14]. It is worth noting that CPAs may not be sufficiently unloaded from the explants during a short period of washing [39]. The DMSO concentration in garlic shoot apices loading (influx) during cryoprotection with PVS2 for 60 min at RT and efflux during unloading with 1.2 M sucrose for 60 min was highly negatively correlated with the moisture content of garlic apices cryoprotected (*r* = −0.961) and unloaded (*r* = −0.962) [98]. The unloading efficiency can be variable; more CPAs are unloaded at a lower sucrose concentration and for a longer duration [39]. The frequent unloading condition is 1.2 M sucrose (41.1%) for 20 min or shorter periods [14]. Consistent with the OS C4-35%, about 35% sucrose (1.02 M) for a longer duration (30–40 min) was proposed for shoot tip vitrification [36].

All through the cryopreservation procedure, including in vitro subculture, excision of shoot tips, PC, OP, CP, C/W (LN), and unloading and regrowth, plant cells and tissues are susceptible to diverse injuries, among them osmotic stress (shock), biochemical toxicity, and freezing injuries (ice crystallization, recrystallization). All these injuries may burst reactive oxygen species (ROS)-mediated oxidative stress [38,99,100] and oxidative stress-related gene expression [101,102]. The oxidative stress gradually increases from the PC, OP, and CP and peaks at C/W (LN), the unloading stage [71,100,103,104,105], and exogenous antioxidants improved LN regeneration [99,100,106,107]. Metabolic rates are the lowest at this stage [108], while total organic acid contents have jumped [105]. Among the 14 organic acids (OAs) tested, four OA (2-hydroxybutyric, 3-hydroxypropionic, lactic, and glycolic) contents were highly correlated (*r* = 0.980) with the CPA concentration employed. It is hypothesized that CPA toxicity is positively correlated with the CPA concentration used, and active modulation of OA metabolism of shoot tips in the DV procedure, from fresh to LN and regrowth, may help them to cope with osmotic stress and chemical cytotoxicity of CPAs [105].

To recover these injured explants, we must incubate them cautiously, including controlled unloading of CPAs, dark conditions, adding growth hormones, and transferring them to a new medium [14]. Initial ammonium-free in regrowth medium 1 (RM1) and growth hormones (gibberellic acid, GA_3_, and benzyl adenine, BA) in regrowth medium 2 (RM2) were crucial for the regeneration of cryoprotected control (LNC) and cryopreserved (LN) shoot tips [7,15,48,69,70,74]. Though ammonium is a vital nitrogen source for plants, ammonium ions in the regrowth medium lead to ROS-induced oxidative stress and inhibit recovery from freezing injury [106,109]. Thus, initially omitting the ammonium ions in the regrowth medium for 5–7 days increased LN regeneration by 61% [15,110]. Therefore, a three-step regrowth medium, initially ammonium-free and with growth hormones (RM1), followed by ammonium-containing and with growth hormones (RM2), and finally, a hormone-free (MSF) medium, is recommended for the sensitive materials. It is worth comparing with the conventional no-transferring regrowth medium to evaluate the ROS-induced oxidative stress.

## 5. A Systematic Approach Balancing the Cryoprotection and Cytotoxicity of CPAs

As DV is a multi-stage procedure from stages 1~5, a systematic approach will optimize each step and tune it as a whole (Table 3). At stage 1 (material preparation), optimization of the in vitro culture system is a prerequisite for preparing vigorous explants and recovery from cryopreservation. One of the purposes of general in vitro culture is propagation efficiency, i.e., as many propagules as possible. But for cryopreservation, the vigor of plantlets is more critical than the multiplication efficiency per se. We could improve DPV by inoculating the apical sections on a Gelrite-gelled medium and overlaying liquid medium during subculture. One or two cycles of a subculture may improve the DPV. Optimization of the in vitro culture system, which is influenced by diverse factors, during the material preparation is a prerequisite for successful cryopreservation. Therefore, more attention is needed to standardize the in vitro material preparation and to select the proper explants.

At stage 2 (pre-LN), for the unknown or sub-optimized species, we can apply a tentative standard protocol for shoot tips: preculture with 10% sucrose (31 h) and 17.5% sucrose (16 h) (two-step preculture), osmoprotect with C4-35% for 30–40 min, and cryoprotect with A3-80% for 60 min at 0 °C. At stages 3 (cooling in LN) and 4 (warming), aluminum foil strip-mounted shoot tips are plunged into LN, stored, rewarmed in the pre-heated (40 °C) 35% sucrose (S-35%), and unloaded for 40 min at RT (solution changed at 15 min). At stage 5 (regrowth), a three-step regrowth initially with RM1 {ammonium (NH_4_)-free regrowth medium} for five days, followed by RM2 (ammonium-containing and growth hormones) for 2–4 weeks and MSF (growth hormone-free) may be applied to regenerate the plantlets (three-step regrowth).

Regrowth of shoot tips on one plate following the tentative standard protocol, i.e., fresh, PC, OP, CP (LNC), and LN, is a valuable tool to characterize the sensitivity of the material and to standardize the procedure by tuning the cryoprotection and cytotoxicity (Figure 1B). The law of diminishing returns in regrowth responses is common [111], and tissues survive cryopreservation but often fail to develop into typical rooted plants [1], which is one of the challenges in plant cryopreservation. One plate in Figure 1B(G + LO) (Gelrite + Liquid-O) is an example of a “soft landing”, a little step-by-step slow growth from fresh, PC, OP, CP, to LN. Even with the same DV protocol, the regrowth of *P. yatabeanus* shoot tips excised from the nodal sections of donor plants grown with G+LX (Gelrite + Liquid-X) was slower or weaker than the G+LO, and only three of five LNC and one of five LN shoots were regenerated (Figure 1B(G + LX)).

One plate of the tentative standard protocol using 20 shoot tips provides valuable lessons for tuning the process. In case the regrowth of PC and afterward is poor, the shoot tips may have lower DPV innately or due to inappropriate subculture conditioning, resulting in slow, slim, short growth in subcultures. In this case, we should improve the DPV in subcultures and test the suitability of a one-step preculture with 10% sucrose. If the regrowth of LNC with A3-80% or B5-85% is quite good, but the LN is significantly lower than the LNC, the shoot tips may tolerate more elevated levels of cryoprotection with A3-90% or PVS3, as summarized in Table 4. Likewise, the one plate helps tune each procedure for more cryoprotection and less cytotoxicity by increasing or decreasing the CPA concentration (primarily) and duration from fresh to LN, resulting in a soft landing.

The optimal concentration of PVS corresponds to the sensitivity to the cytotoxicity of PVS. In comparison, the incubation time correlates with the size and permeability of the explants at both 0 °C or RT. Therefore, for the cryoprotection of normal-size shoot tips, it is recommended to test the concentration of PVS (A3-90% vs. A3-80% at 0 °C, PVS3 vs. B5-85% at RT) rather than the incubation time at the initial stage; 60 min was acceptable with diverse species [7,15,42,48,53,54,56,62,63,66,68,69,70]. If the material is incompatible with 60 min cryoprotection, it might have lower DPV innately. Moreover, the material preparation from subcultures to preconditioning is often under-optimized.

If more explants are available, in addition to the tentative standard protocol, additional options such as 10% sucrose preculture (2 days), cryoprotection with PVS2 (60 min, 0 °C), B5-85% (60 min, RT), and regrowth with RM2~ (no transfer) can be tested, which make up to five conditions. We may characterize the material using this mini set and tune the standard protocol. We can examine the factors after establishing the standard protocol or approximately optimizing the process through the preliminary investigation. If not, we may not correctly analyze the elements, like in the case of *P. yatabeanus* [15] and *P. chinense* [48]. All the treatment conditions produced non-significantly below 21% LN regeneration for the former, and the latter resulted in misleading conclusions of A- and B-series PVS with lower and less-significant LN regeneration.

## 6. Conclusions

DV is a multi-stage procedure with many factors, from material preparation to regrowth. Since the cytotoxicity of CPA mixtures (PVS) is a primary limiting factor in DV approaches, DPV is crucial for coping with the toxicity of PVS. The DPV is innate and can be maximized with appropriate material preparations, i.e., vigorously growing in subcultures aided by a liquid overlay on top of the gelled medium, selecting proper explants, and a two-step preculture. Developing the DV protocol starts with testing the material with a tentative standard protocol, which is composed of a two-step preculture {10% sucrose (31 h) and 17.5% sucrose (16 h)}, osmoprotection with C4-35%, cryoprotection with A3-80% (60 min at 0 °C), cooling, and rewarming using aluminum foil strips.

Using a three-step regrowth initially with ammonium-free regrowth medium for five days, regrowth of a total of 20 shoot tips in one plate following the successive stages of a tentative standard protocol, i.e., fresh, PC, OP, LNC, and LN, is a valuable tool to characterize the sensitivity of the material and to standardize the procedure by tuning the cryoprotection and cytotoxicity of CPAs.

A-series PVS (A3-90%, A3-80%) and B-series PVS (PVS3, B5-85%) can be tested based on the DPV. For micro-sized, soft, and susceptible materials, A3-70% and B5-80% can be an option. These alternative PVSs have been applied in over 30 pieces of literature with an 8.5~67.3% increase in LN regeneration compared to PVS2 and PVS3 treatments. Using this approach as an alternative to blind condition screening would be influential in broadening cryopreservation to diverse wild species and problem materials.

## Figures and Tables

**Table 2:** Cryopreservation studies using variants of PVS2 and PVS3 and comparison with their original PVS in chronological order from 2009 to 2023.

Species (Explants ^1^)	(Method ^2^) Sucrose Concentration (Hours)	Osmo- and Cryo-Protection ^3^	LN Regrowth (R) or Regeneration (RG) (+/−% Compared to PVS2) ^4^	References
*Allium sativum* (clove apices, 3.5 × 3 mm)	(DV) S-10% (3 d, Solid medium)	G18.4% + S20.5% (50 min) → PVS2 (30 min, RT)	LNC-RG: 99.0% LN-RG: 50.2%	[44]
		G18.4% + S20.5% (50 min) → A3-90% (30 min, RT)	LNC-RG: 97.9% LN-RG: 69.9% (+19.7%)	
		G18.4% + S20.5% (50 min) → PVS3 (150 min, RT)	LNC-RG: 97.9% LN-RG: 98.8% (+48.6%)	
*Chrysanthemum morifolium* (apical ST, 1.2–1.5 mm)	(DV) S-10% (27 h) → S-17.5% (18 h) → S-25% (8 h)	G18.4% + S20.5% (40 min) → PVS2 (20 min, RT)	LNC-RG: 75.9% LN-RG: 30.8%	
		G18.4% + S20.5% (40 min)→ A3-90% (20 min, RT)	LNC-RG: 75.3% LN-RG: 59.8% (+29.0%)	
		G18.4% + S20.5% (40 min) → PVS3 (60 min, RT)	LNC-RG: 86.7% LN-RG: 73.1% (+42.3%)	
*Chrysanthemum morifolium* (apical ST, 1.2–1.5 mm)	(DV) S-10% (27 h) → S-17.5% (18 h) → S-25% (8 h)	C7-32.1% (40 min) → PVS3 (60 min, RT)	RG: 53.9%	[42]
		G9.2% + S24.0% (40 min) → PVS3 (60 min, RT)	RG: 39.0%	
		C4-35% (40 min) → PVS3 (60 min, RT)	RG: 65.3% (+11.4%)	
*Rubia akane* (hairy roots)	(DV) S-10% (24 h) → S-17.5% (5 h)	C4-35% (20 min) → PVS2 (10 min, RT)	R: 19.0%	[41]
		C4-35% (20 min) → A3-80% (10 min, RT)	R: 27.5% (+8.5%)	
		C4-35% (20 min) → A3-70% (10 min, RT)	R: 43.2% (+24.2%)	
	(DV) S-10% (54 h) → S-17.5% (5 h)	C7-32.1% (20 min) → B5-80% (20 min, RT)	R: 57.1% (+38.1%)	
		C4-35% (20 min) → B5-80% (20 min, RT)	R: 72.4% (+53.4%)	
		C4-35% (30 min) → A3-70% (20 min, ice)	R: 83.8% (+64.8%)	
		C4-35% (30 min) → B5-80% (15 min, RT)	R: 86.3% (+67.3%)	
*Chrysanthemum morifolium* (axillary ST)	(DV) S-10% (31 h) → S-17.5% (17 h) → S-25% (7 h)	C4-35% (40 min) → PVS2 (25 min, RT)	RG: 30.8%	[59]
		C4-35% (40 min) → A7-90% (25 min, RT)	RG: 65.3% (+34.5%)	
		C4-35% (40 min) → PVS3 (60 min, RT)	RG: 70.9% (+40.1%)	
		C4-35% (40 min) → B5-80% (60 min, RT)	RG: 55.8% (+25.0%)	
*Rubus fruticisus* (ST, 1–2 mm)	(DV) S-10% (15 h) → S-25% (5 h)	G17.5%+S17.1% (30 min)→ A3-90% (30 min, RT)	R: 30.0%	[60]
		G17.5%+S17.1% (30 min) → PVS3 (60 min, RT)	R: 70.0% (+40.0%)	
*Prunus cerasifera* (ST, 1–2 mm)	(DV) S-10% (15 h) → S-25% (5 h)	G17.5%+S17.1% (30 min)→ A3-90% (10 min, RT)	R: 20.0%	
		G17.5%+S17.1% (30 min) → PVS3 (90 min, RT)	R: 15.0%	
*Rubia akane* (hairy roots)	(DV) S-10% (48 h) → S-17.5% (4 h)	C7-32.1% (20 min) → B5-80% (15 min, RT)	R: 51.1%	[45]
		C4-35% (20 min) → B5-80% (15 min, RT)	R: 72.4% (+21.3%)	
		C4-35% (20 min) → PVS2 (20 min, 0 °C)	R: 73.3%	
		C4-35% (20 min) → A3-70% (20 min, 0 °C)	R: 83.8% (+10.5%)	
		C4-35% (20 min) → B5-80% (15 min, RT)	R: 82.0% (+8.7%)	
*Kalopanax septemlobus* (embryogenic callus)	(Vit) S-10% (17 h) → S-17.5% (3 h) → S-25% (3 h)	C6-40% (20 min) → PVS2 (20 min, 0 °C)	R: 94.6%	[47]
		C6-40% (20 min) → A3-80% (40 min, 0 °C)	R: 99.3%	
		C6-40% (20 min) → B3-90% (15 min, RT)	R: 92.5%	
*Solanum tuberosum* (potato; ST)	(DV) S-10% (7 h) → S-25% (17 h)	No-loading → PVS2 (20 min, RT)	RG: 21.7%	[61]
		No-loading → A7-90% (30 min, RT)	RG: 80.9% (+59.2%)	
		No-loading → PVS3 (90 min, RT)	RG: 57.2% (+35.5%)	
*Lithodora rosmarinifolia* (apical node, 2–3 mm)	(DV) S-10% (16 h) → S-25% (5 h)	C4-35% (20 min) → A3-90% (30 min, RT)	R: 17%	[62]
		C4-35% (20 min) → PVS3 (60 min, RT)	R: 33% (+16%)	
		C4-35% (20 min) → B5-80% (120 min, RT)	R: 23%	
*Prunus cerasus* × *P. canescens* ‘Gisela 5′ (ST 1–2 mm)	(DV) S-10% (15 h) → S-25% (5 h)	G17.5% + S17.1% (30 min) → A3-90% (40 min, RT)	R: 38.2%	[63]
		G17.5% + S17.1% (30 min) → PVS3 (60 min, RT)	40.0%	
*Lilium* spp. (bulblets, 2 mm)	(DV) S-10% (31 h) → S-25% (16 h)	C4-35% (40 min) → PVS2 (90 min, RT)	RG: 18.3%	[64]
		C4-35% (40 min) → A3-90% (90 min, RT)	RG: 32.3% (+14%)	
		C4-35% (40 min) → PVS3 (240 min, RT)	RG: 87.5% (+69.2%)	
*Prunus cerasus* × *P. canescens* ‘Gisela 5′ (ST, 2 mm)	(Vit) S-10% (15 h) → S-17.5% (5 h)	C7-32.1% (20 min) → PVS2 (30–50 min, 0 °C)	R: 8-17%	[51]
		C7-32.1% (20 min) → A3-90% (30–50 min, 0 °C)	R: 39–56% (+31~39%)	
*Rubia akane* (hairy roots, 1.5–2.0 mm)	(EV) Encapsule →S-10% (72 h)→ S-17.5% (24 h)	C6-40% (50 min) → A3-90% (40 min, 0 °C)	R: 97.5%	[49]
*Rubia akane* (hairy roots)	(DV) S-10% (48 h) → S-17.5% (4 h)	C4-35% (30 min) → A3-70% (20 min, 0 °C)	R: 83.8%	[65]
*Prunus domestica* “Sitnica” (ST)	(DV) (no information on preculture)	G17.5% + S17.1% (30 min)→ A3-90% (30 min, RT)	R: 10.0%	[66]
		G17.5% + S17.1% (30 min) → PVS3 (90 min, RT)	R: 18.2%	
*Prunus insititia* “Crvena Ranka” (ST)	(DV) (no information on preculture)	G17.5% + S17.1% (30 min) → A3-90% (20~30 min, RT)	R: 36.4%	
		G17.5% + S17.1% (30 min) → PVS3 (60 min, RT)	R: 30.0%	
*Prunus cerasifera* (ST)	(V Cryo-plate) S-10% (24 h) → gel	C7-32.1% (30 min) → A3-90% (30 min, RT)	R: 56.1%	[55]
		G17.5% + S17.1% (30 min) → A3-90% (30 min, RT)	R: 41.7%	
	(D Cryo-plate) S-10% (24 h) → gel	G17.5% + S17.1% (30 min) → air-current (2–3 h)	R: 57.7–77.5%	
*Prunus domestica* ‘Pozegaca’ (ST)	(V Cryo-plate) S-10% (24 h) → gel	C7-32.1% (30 min) → A3-90% (30 min, RT)	R: 44.6%	
		G17.5% + S17.1% (30 min) → A3-90% (30 min, RT)	R: 34.2%	
	(D Cryo-plate) S-10% (24 h) → gel	G17.5% + S17.1% (30 min) → air-current (2–3 h)	R: 28.5–47.5	
*Clinopodium odorum* (ST, 1 mm)	(V Cryo-plate) S-10% (17 h) → gel	C7-32.1% (20 min) → PVS2 (60 min, 0 °C)	RG: 50.2%	[56]
		C-Nii-38.9% (20 min) → PVS2 (60 min, 0 °C)	RG: 27.2%	
		C4-35% (20 min) → PVS2 (60 min, 0 °C)	RG: 57.4% (+6.8%)	
		C6-40% (20 min) → PVS2 (60 min, 0 °C)	RG: 36.5%	
		C7-32.1% (20 min) → PVS2 (60 min, 0 °C)	RG: 36.2%	
		C7-32.1% (20 min) → A3-90% (60 min, 0 °C)	RG: 22.4% (−13.8%)	
		C7-32.1% (20 min) → PVS3 (60 min, RT)	RG: 34.4%	
*Betula lenta* (ST, 1.5–2.0 mm)	(EV) encapsulation → S-10% (24 h)	C4-35% (20 min) → A3-90% (80 min, 0 °C)	RG: 13%	[54]
		C7-32.1% (20 min) → PVS3 (60 min, RT)	RG: 0%	
	(DV) S-10% (24 h)	C4-35% (20 min) → PVS3 (60 min, RT)	RG: 13%	
*Rubus fruticosus* (ST, 1–2 mm)	(DV) S-10% (15 h) → S-25% (5 h)	C4-35% (30 min) → A3-90% (40 min, ice)	RG: 55-65%	[67]
		C4-35% (30 min) → PVS3 (40 min, RT)	RG: 80–90% (+15~35%)	
*Castilleja levisecta* (ST)	(DV) S-10% (17 h) → S-17.5% (4 h)	C4-35% (40 min) → A3-90% (40 min, ice)	R: 66%	[68]
		C4-35% (40 min) → PVS3 (60 min, RT)	R: 70%	
*Chrysanthemum morifolium* (ST)	(DV) S-10% (30 h) → S-17.5% (16 h)	C6-40% (30 min) → B5-80% (60 min, RT)	R: 80.7%	[69]
*Aster altaicus* (ST, 1.5 mm)	(DV) S-10% (55 h) → S-17.5% (17 h)	C4-35% (30 min, 25 °C) → PVS2 (20 min, RT)	RG: 38.8%	[70]
		C4-35% (30 min, 25 °C) → A3-90% (20 min, RT)	RG: 54.5% (+15.7%)	
		C4-35% (60 min, ice) → A3-80% (60 min, ice)	RG: 65.3% (+26.5%)	
		C4-35% (30 min, 25 °C) → PVS3 (60 min, RT)	RG: 35.0%	
		C4-35% (30 min, 25 °C) → B5-80% (60 min, RT)	RG: 52.0% (+13.2%)	
*Panax ginseng* (adventitious roots)	(DV) S-10% (24 h)	C4-35% (20 min) → A3-90% (10 min, RT)	R: 5%	[71]
		C4-35% (20 min) → B5-80% (10 min, RT)	R: 15%	
*Fragaria* × *ananassa* (2 var., ST, 1–2 mm)	(DV) S-10% (40 h)	C4-35% (40 min) → B5-80% (40 min, RT)	R: 50.5~55.6%	[72]
*Dendrobium moniliforme* (seeds)	(Vit) S-10% (31 h) → S-17.5% (17 h)	C4-35% (30 min) → A3-90% (60 min, 0 °C)	Germination: 74%	[52]
		C4-35% (30 min) → PVS3 (30 min, RT)	Germination: 80%	
*Cymbibidium kanran* (rhizomes)	(DV) S-17.5% (31 h) → S-25% (17 h)	C4-35% (15 min) → C11-60% (25 min) → PVS3 (90 min, RT)	R: 90%	
*Hypericum perforatum* (roots)	(DV) S-10% (17 h)	C4-35% (20 min) → B5-80% (30 min, RT)	R: 78%	[73]
*Prunus domestica* var. ‘Crvena Rankat’ (ST)	(V cryo-plate) S-10% (24 h) → gel	C4-35% (30 min) → A3-90% (40 min, RT)	R: 51.9%	[57]
		C4-35% (30 min) → PVS3 (60 min, RT)	R: 66.7%	
	(D cryo-plate) S-10% (24 h) → gel	C4-35% (30 min) → air-current (2–3 h)	R: 30-40%	
*Malus* × *domestica* ‘Gala Must’ (ST)	(Vit) S-10% (15 h) → S-25% (5 h)	C7-32.1% (20 min) → PVS2 (50 min, 0 °C)	R: 40%	[53]
		C7-32.1% (20 min) → A3-90% (50 min, 0 °C)	R: 75% (+35.0%)	
*Pogostemon yatabeanus* (ST, 1.5 mm)	(DV) S-10% (31 h)	C4-35% (40 min) → PVS2 (60 min, 0 °C)	RG: 0.0%(NH_4_-containing RM2)	[15]
		C4-35% (40 min) → A3-90% (60 min, 0 °C)	RG: 0.0% (RM2)	
		C4-35% (40 min) → A3-80% (60 min, 0 °C)	RG: 17.7% (+17.7%) (RM2)	
*Pogostemon yatabeanus* (ST, 1.5 mm)	(DV) S-10% (31 h)	C4-35% (40 min) → A3-80% (60 min, 0 °C)	RG: 88.2% (RM1)	[74]
		C4-35% (40 min) → B5-80% (60 min, RT)	RG: 77.8% (RM1)	
*Lupinus rivularis* (apical ST, 1.5 mm)	(DV) S-10% (24 h)	C4-35% (20 min) → A3-90% (30 min, ice)	RG: 62%	[75]
*Penthorum chinense* (ST, 1.3 mm)	*Conventional donor plants* (7 w) → (DV) S-10% (31 h) → S-17.5% (17 h)	C4-35% (20 min) → PVS2 (30 min, 0 °C)	RG: 41.1%	[48]
		C4-35% (20 min) → A3-80% (30 min, 0 °C)	RG: 36.8%	
		C4-35% (20 min) → A3-70% (30 min, 0 °C)	RG: 52.3% (+11.2%)	
		C4-35% (20 min) → B5-80% (30 min, RT)	RG: 40.9%	
	*Rejuvenized donor plants* (5–6 w) → (DV) S-10% (31 h) → S-17.5% (17 h)	C4-35% (40 min) → PVS2 (60 min, 0 °C)	RG: 45.5%	
		C4-35% (40 min) → A3-80% (60 min, 0 °C)	RG: 53.6% (+8.1%)	
		C4-35% (40 min) → B5-85% (60 min, RT)	RG: 64.2% (+18.7%)	
*Chrysanthemum morifolium* (ST)	(DV) S-10% (31 h) → S-17.5% (16 h)	C4-35% (40 min) → PVS2 (60 min, 0 °C)	RG: 66.2%	[7]
		C4-35% (40 min) → A3-90% (60 min, 0 °C)	RG: 56.7%	
		C4-35% (40 min) → A3-80% (60 min, 0 °C)	RG: 85.4% (+19.2%)	
		C4-35% (40 min) → PVS3 (60 min, RT)	RG: 43.9%	
		C4-35% (40 min) → B5-85% (60 min, RT)	RG: 78.8% (+12.6%)	
*Prunus domestica* var. ‘Crvena Rankat’ (ST)	(V cryo-plate) S-10% (24 h) → gel	C4-35% (30 min) → PVS2 (40 min, RT)	R: 0~33.3%	[58]
		C4-35% (30 min) → A3-90% (40 min, RT)	R: 0~62.5%	
		C4-35% (30 min) → PVS3 (80 min, RT)	R: 8.3~45.8%	
	(D cryo-plate) S-10% (24 h) → gel	C7-32.1% (30 min) → air-current (2.5 h)	R: 0~55.0%	

^1^ ST, shoot tips; ^2^ Vit, vitrification; DV, droplet vitrification; EV, encapsulation–vitrification; V-plate, V cryo-plate; D-plate, D cryo-plate; ^3^ G, glycerol; DMSO, dimethyl sulfoxide; EG, ethylene glycol; S, sucrose; C4-35%, G 17.5% + S 17.5% [42]; C6-40%, G 20% + S 20% [42]; PVS2, G 30% + DMSO 15% + EG 15% + S 13.7% [3]; A3-90%, G 37.5% + DMSO 15% + EG 15% + S 22.5% [44]; A3-80%, G 33.3% + DMSO 13.3% + EG 13.3% + S 20.1% [47]; A3-70%, G 29.2% + DMSO 11.7% + EG 11.7% + S 17.4% [41]; A7-90%, G 37.5% + DMSO 10.0% + EG 10.0% + S 32.5% [44]; PVS3, G 50% + S 50% [43]; B3-90%, G 45% + S 45% [44]; B5-85%, G 42.5% + S 42.5% [48]; B5-80%, G 40% + S 40% [44]; RT, room temperature; ^4^ regrowth (R), and regeneration (RG) before (LNC) and after (LN) cryopreservation using the standard protocol reported.

**Table 3:** A systematic approach for balancing the cryoprotection and cytotoxicity of CPAs during the droplet-vitrification procedure.

Stages	1 (Material)	2 (Pre-LN) ^1^	3 (LN)	4 (Warming)	5 (Regrowth) ^2^
Procedure	- Initiation and subculture	- Preculture - Osmoprotection - Cryoprotection	- Cooling	- Rewarming - Unloading	- Survival - Regeneration
Key points (parameter)	- Donor plant vigor (rapid growth, dry weight) - Proper explants	- Osmotic adaptation - Balancing cryoprotection and cytotoxicity	- Cooling velocity	- Warming velocity - Removal of CPAs	- Soft landing - Special care to reduce oxidative stress
Options to test	- Optimization of in vitro culture system - Apical section and liquid overlay - Preconditioning and selecting proper explants (position, stage, size)	- Two-step preculture (S-10%, 31 h → S-17.5% 16 h)/One step (S-10%, 24 h) - C4-35%, 30–40 min - A3-80% (0 °C, 60 min)	- Cooling containers	- Warming in pre-heated (40 °C) unloading solution (S-35%) and unloading for 40 min (solution change at 15 min)	- Three-step regrowth (initially ammonium-free for 5 days → with growth hormones → lastly growth hormone-free for regeneration) - Regrowth “one-plate” following each stage (fresh, PC, OP, CP, LN)

^1^ S-10%, sucrose 10% (0.3 M); S-17.5%, sucrose 17.5% (0.5 M); C4-35%, G 17.5% + S 17.5% [42]; A3-80%, G 33.3% + DMSO 13.3% + EG 13.3% + S 20.1% [47]; G, glycerol; DMSO, dimethyl sulfoxide; EG, ethylene glycol; S, sucrose. ^2^ fresh, non-treated control; PC, preculture only; OP, preculture and osmoprotection (PC-OP); CP, preculture, osmoprotection, cryoprotection, and unloading (PC-OP-CP-UL); LN, preculture, osmoprotection, cryoprotection, cooling, rewarming, and unloading (PC-OP-CP-LN-UL).

**Table 4:** Tentative standard protocol for three categories of in vitro plant materials in droplet-vitrification method.

Material	Explants	Preculture ^1^	Osmoprotection ^2^	Cryoprotection ^3^
Micro, soft	Callus, Roots	S-10% (48 h)	C4-35% (20 min)	PVS2, A3-70%/B5-80% (~30 min)
Shoot tips	Sensitive (small-sized)	S-10% (48 h)	C4-35% (30 min)	A3-70%/B5-80% (60 min)
	Moderate (medium)	S-10% (31 h) → S-17.5% (16 h)	C4-35% (30 min)	A3-80%/B5-85% (60 min)
	Tolerant (large)	S-10% (31 h) → S-17.5% (16 h)	C4-35% (40 min)	A3-90%/PVS3 (60 min)
Hard-structured	Bulbs, Embryos	S-17.5% (31 h) → S-25% (16 h)	C4-35% (40 min)	A3-90%/PVS3 (120 min)

^1^ S-10%, sucrose 10% (0.3 M); S-17.5%, sucrose 17.5% (0.5 M); ^2^ C4-35%, G 17.5% + S 17.5% [42]; ^3^ PVS2, G 30% + DMSO 15% + EG 15% + S 13.7% [3]; A3-90%, G 37.5% + DMSO 15% + EG 15% + S 22.5% [44]; A3-80%, G 33.3% + DMSO 13.3% + EG 13.3% + S 20.1% [47]; A3-70%, G 29.2% + DMSO 11.7% + EG 11.7% + S 17.4% [41]; PVS3, G 50% + S 50% [43]; B5-85%, G 42.5% + S 42.5% [48]; B5-80%, G 40% + S 40% [44]; G, glycerol; DMSO, dimethyl sulfoxide; EG, ethylene glycol; S, sucrose. Cryoprotection with A-series at 0 °C; B-series at room temperature (RT).

## Data Availability

Not applicable.

## References

[1] Panis B. (2019). Sixty years of plant cryopreservation: From freezing hardy mulberry twigs to establishing reference crop collections for future generations. Acta Hortic..

[2] Pence V.C., Ballesteros D., Walters C., Reed B.M., Philpott M., Dixon K.W., Pritchard H.W., Culley T.M., Vanhove A.C. (2020). Cryobiotechnologies: Tools for expanding long-term ex situ conservation to all plant species. Biol. Conserv..

[3] Sakai A., Kobayashi S., Oiyama I. (1990). Cryopreservation of nucellar cells of naval orange (*Citrus sinensis* Osb. var. *brasiliensis* Tanaka) by vitrification. Plant Cell Rep..

[4] Dereuddre J., Scottez C., Arnaud Y., Duron M. (1990). Resistance of alginate-coated axillary shoot tips of pear tree (*Pyrus communis* L. cv. Beurre Hardy) in vitro plantlets to dehydration and subsequent freezing in liquid nitrogen. C. R. Acad. Sci. Paris.

[5] Panis B., Piette B., Swennen R. (2005). Droplet vitrification of apical meristems: A cryopreservation protocol applicable to all Musa- ceae. Plant Sci..

[6] Acker J.P., Adkins S., Alves A., Horna D., Toll J. (2017). Feasibility study for a safety back-up cryopreservation facility. Independent Expert Report: July 2017.

[7] Lee H., Park J., Park S.-U., Kim H. (2023). Alternative plant vitrification solution A3-80% and initial ammonium-free regrowth medium enable cryobanking of chrysanthemum germplasm. Plants.

[8] Reed B.M., Kovalchuk I., Kushnarenko S., Meier-Dinkel A., Schoenweiss K., Pluta S., Straczynska K., Benson E.E. (2004). Evaluation of critical points in technology transfer of cryopreservation protocols to international plant conservation laboratories. CryoLetters.

[9] Baalbaki R.Z.B., Association of Official Seed Analysts (AOSA) (2009). Seed Vigor Testing Handbook.

[10] Matsumoto T., Sakai A., Takahashi C., Yamada K. (1995). Cryopreservation of apical meristems of wasabi (*Wasabia japonica*) by encapsulation-vitrification method. CryoLetters.

[11] Yamamoto S., Rafique T., Priyantha W.S., Fukui K., Matsumoto T., Niino T. (2011). Development of a cryopreservation procedure using aluminium cryo-plates. CryoLetters.

[12] Niino T., Yamamoto S., Fukui K., Castillo-Martinez C.R., Matsumoto T., Engelmann F. (2013). Dehydration improves cryopreservation of mat rush (*Juncus decipiens* Nakai) basal stem buds on cryo-plates. CryoLetters.

[13] Benson E.E. (2008). Cryopreservation of phytodiversity: A critical appraisal of theory & practice. Crit. Rev. Plant Sci..

[14] Popova E., Kulichenko I., Kim H.-H. (2023). Critical role of regrowth conditions in post-cryopreservation of in vitro plant germplasm. Biology.

[15] Lee H., Park H., Popova E., Lee Y.Y., Park S.U., Kim H.H. (2021). Ammonium-free medium is critical for regeneration of shoot tips of the endangered species *Pogostemon yatabeanus* cryopreserved using droplet-vitrification. CryoLetters.

[16] Wowk B. (2010). Thermodynamic aspects of vitrification. Cryobiology.

[17] Fahy G.M., MacFarlane D.R., Angell C.A., Meryman H.T. (1984). Vitrification as an approach to cryopreservation. Cryobiology.

[18] Teixeira A.S., González-Benito M.E., Molina-García A.D. (2014). Measurement of cooling and warming rates in vitrification-based plant cryopreservation protocols. Biotechnol. Prog..

[19] Wowk B. (2013). Metastable vitrification of cryoprotectant solutions. Cryobiology.

[20] Fahy G.M., Wowk B., Wu J., Paynter S. (2004). Improved vitrification solutions based on the predictability of vitrification solution toxicity. Cryobiology.

[21] Fahy G.M., Wowk B. (2015). Principles of cryopreservation by vitrification. Methods Mol. Biol..

[22] Kim H.H., Yoon J.W., Park Y.E., Cho E.G., Sohn J.K., Kim T.S., Engelmann F. (2006). Cryopreservation of potato cultivated and wild species: Critical factors in droplet vitrification. CryoLetters.

[23] Kim H.H., Lee J.K., Yoon J.W., Ji J.J., Nam S.S., Hwang H.S., Cho E.G., Engelmann F. (2006). Cryopreservation of garlic bulbil primordia by the droplet-vitrification procedure. CryoLetters.

[24] Zhao G., Fu J. (2017). Microfluidics for cryopreservation. Biotechnol. Adv..

[25] Seki S., Mazur P. (2009). The dominance of warming rate over cooling rate in the survival of mouse oocytes subjected to a vitrification procedure. Cryobiology.

[26] Tao D., Li P.H. (1986). Classification of plant cell cryoprotectants. J. Theor. Biol..

[27] Raju R., Bryant S.J., Wilkinson B.L., Bryant G. (2021). The need for novel cryoprotectants and cryopreservation protocols: Insights into the importance of biophysical investigation and cell permeability. Biochim. Et Biophys. Acta (BBA)-Gen. Subj..

[28] Fahy G.M. (1984). Cryoprotectant toxicity biochemical or osmotic. CryoLetters.

[29] Chang T., Zhao G. (2021). Ice inhibition for cryopreservation: Materials, strategies, and challenges. Adv. Sci..

[30] Zamecnik J., Faltus M., Bilavcik A. (2021). Vitrification solutions for plant cryopreservation: Modification and properties. Plants.

[31] Zhang M., Gao C., Ye B., Tang J., Jiang B. (2019). Effects of four disaccharides on nucleation and growth of ice crystals in concentrated glycerol aqueous solution. Cryobiology.

[32] Salisbury F.R., Ross C.W. (1991). Plant Physiology.

[33] Volk G.M., Walters C. (2006). Plant vitrification solution 2 lowers water content and alters freezing behavior in shoot tips during cryoprotection. Cryobiology.

[34] Warner R.M., Brown K.S., Benson J.D., Eroglu A., Higgins A.Z. (2022). Multiple cryoprotectant toxicity model for vitrification solution optimization. Cryobiology.

[35] Elliott G.D., Wang S., Fuller B.J. (2017). Cryoprotectants: A review of the actions and applications of cryoprotective solutes that modulate cell recovery from ultra-low temperatures. Cryobiology.

[36] Kim H.H., Popova E. (2023). Unifying principles of cryopreservation protocols for new plant materials based on alternative cryoprotective agents (CPAs) and a systematic approach. CryoLetters.

[37] Mazur P., Leibo S.P., Chu E.H.Y. (1972). A two-factor hypothesis of freezing injury: Evidence from Chinese hamster tissue culture cells. Exp. Cell Res..

[38] Best B.P. (2015). Cryoprotectant toxicity: Facts, issues, and questions. Rejuvenation Res..

[39] Kim J.B., Kim H.H., Baek H.J., Cho E.G., Kim Y.H., Engelmann F. (2005). Changes of sucrose and glycerol concentration in garlic shoot tips during freezing using PVS3 solution. CryoLetters.

[40] Salma M., Engelmann-Sylvestre I., Collin M., Escoute J., Lartaud M., Yi J.Y., Kim H.H., Verdeil J.L., Engelmann F. (2014). Effect of the successive steps of a cryopreservation protocol on the structural integrity of *Rubia akane* Nakai hairy roots. Protoplasma.

[41] Kim H.H., Popova E.V., Yi J.Y., Cho G.T., Park S.U., Lee S.C., Engelmann F. (2010). Cryopreservation of hairy roots of *Rubia akane* (Nakai) using a droplet-vitrification procedure. CryoLetters.

[42] Kim H.H., Lee Y.G., Park S.U., Lee S.C., Baek H.J., Cho E.G., Engelmann F. (2009). Development of alternative loading solutions in droplet-vitrification procedures. CryoLetters.

[43] Nishizawa S., Sakai A., Amano Y., Matsuzawa T. (1993). Cryopreservation of asparagus (*Asparagus officinalis* L.) embryogenic suspension cells and subsequent plant regeneration by vitrification. Plant Sci..

[44] Kim H.H., Lee Y.G., Shin D.J., Ko H.C., Gwag J.G., Cho E.G., Engelmann F. (2009). Development of alternative plant vitrification solutions in droplet-vitrification procedures. CryoLetters.

[45] Kim H.H., Popova E.V., Shin D.J., Bae C.H., Baek H.J., Park S.U., Engelmann F. (2012). Development of a droplet-vitrification protocol for cryopreservation of *Rubia akane* (Nakai) hairy roots using a systematic approach. CryoLetters.

[46] Murray K.A., Gibson M.I. (2022). Chemical approaches to cryopreservation. Nat. Rev. Chem..

[47] Shin D.J., Kong H., Popova E.V., Moon H.K., Park S.Y., Park S.U., Lee S.C., Kim H.H. (2012). Cryopreservation of *Kalopanax septemlobus* embryogenic callus using vitrification and droplet-vitrification. CryoLetters.

[48] Zilani R.A.K.M., Lee H., Popova E., Kim H. (2022). In vitro multiplication and cryopreservation of *Penthorum chinense* shoot tips. Life.

[49] Shin D.J., Lee H.E., Bae C.H., Park S.U., Kang H.N., Kim H.H. (2014). Development of an encapsulation-vitrification protocol for *Rubia akane* (nakai) hairy roots: A comparison with non-encapsulation. CryoLetters.

[50] Sakai A., Engelmann F. (2007). Vitrification, encapsulation-vitrification and droplet-vitrification. CryoLetters.

[51] Ružić D., Vujović T., Cerović R. (2014). Cryopreservation of cherry rootstock Gisela 5 using vitrification procedure. Hort. Sci..

[52] Popova E., Kim H.H. (2019). Development of cryopreservation protocols for endangered wild orchids in Korea. Acta Hortic..

[53] Vujović T., Ružić Đ., Cerović R. (2021). Cryopreservation of apple shoot tips by vitrification and subsequent plant regeneration. Acta Hortic..

[54] Rathwell R., Popova E., Shukla M.R., Saxena P.K. (2016). Development of cryopreservation methods for cherry birch (*Betula lenta* L.), an endangered tree species in Canada. Can. J. For. Res..

[55] Vujović T., Chatelet P., Ružić D., Engelmann F. (2015). Cryopreservation of *Prunus* spp. using aluminium cryo-plates. Sci. Hortic..

[56] Engelmann-Sylvestre I., Engelmann F. (2015). Cryopreservation of *in vitro*-grown shoot tips of *Clinopodium odorum* using aluminium cryo-plates. In Vitro Cell. Dev. Biol.-Plant.

[57] Vujović T., Jevremović D., Marjanović T., Ružić Đ. (2021). Cryopreservation of Serbian autochthonous plum ‘Crvena Ranka’ using aluminium cryo-plates. Genetika.

[58] Vujović T., Anđelić T., Vasilijević B., Jevremović D., Engelmann F. (2023). Cryopreservation of indigenous plums and monitoring of multiplication and rooting capacity of shoots obtained from cryopreserved specimens. Plants.

[59] Lee Y.G., Popov E., Cui H.Y., Kim H.H., Park S.U., Bae C.H., Lee S.C., Engelmann F. (2011). Improved cryopreservation of chrysanthemum (*Chrysanthemum morifolium*) using droplet-vitrification. CryoLetters.

[60] Vujović T., Sylvestre I., Ružić D., Engelmann F. (2011). Droplet-vitrification of apical shoot tips of *Rubus fruticosus* L. and *Prunus cerasifera* Ehrh. Sci. Hortic..

[61] Yi J.Y., Lee S.Y., Lee G.A., Jeong J.W., Cho J.H., Kim H.H. (2012). Improvement of the droplet-vitrification method for the cryopreservation of cultivated potato shoot tips. Kor. J. Breed. Sci..

[62] Barraco G., Sylvestre I., Iapichino G., Engelmann F. (2013). Investigating the cryopreservation of nodal explants of *Lithodora rosmarinifolia* (Ten.) Johnst., a rare, endemic Mediterranean species. Plant Biotechnol. Rep..

[63] Ružić D., Vujović T., Cerović R. (2013). Cryopreservation of cherry rootstock Gisela 5 (*Prunus cerasus* × *Prunus canescens*) shoot tips by droplet-vitrification technique. J. Hortic. Res..

[64] Yi J.Y., Lee G.A., Jeong J.W., Lee S.Y., Lim K.B. (2013). Efficient cryopreservation of *Lilium* spp. shoot tips using droplet-vitrification. Plant Breed. Biotech..

[65] Park S.U., Kong H.J., Shin D.J., Bae C.H., Lee S.C., Bae C.H., Rha E.S., Kim H.H. (2014). Development of vitrification protocol in *Rubia akane* (Nakai) hairy roots using a systematic approach. CryoLetters.

[66] Vujović T.I., Ružić Đ.V., Cerović R.M. (2015). Cryopreservation of Serbian autochthonous *Prunus* spp. by droplet-vitrification. Biologia.

[67] Vujović T., Ružić Đ., Cerović R. (2017). Effect of the duration of liquid nitrogen storage on the regrowth of blackberry cryopreserved by droplet vitrification. Contemp. Agric..

[68] Salama A., Popova E., Jones M.P., Shukla M.R., Fisk N.S., Saxena P.K. (2018). Cryopreservation of the critically endangered golden paintbrush (*Castilleja levisecta* Greenm.): From nature to cryobank to nature. Vitr. Cell. Dev. Biol.-Plant.

[69] Yi J.Y., Balaraju K., Baek H.J., Yoon M.S., Kim H.H., Lee Y.Y. (2018). A successful regeneration from shoot tips of *Chrysanthemum morifolium* (Ramat.) following cryopreservation by droplet-vitrification. Korean J. Plant Res..

[70] Choi C.H., Popova E., Lee H., Park S.U., Ku J., Kang J.H., Kim H.H. (2019). Cryopreservation of endangered wild species, *Aster altaicus* var. *uchiyamae* Kitam, using droplet-vitrification procedure. CryoLetters.

[71] Le K.C., Kim H.H., Park S.Y. (2019). Modification of the droplet-vitrification method of cryopreservation to enhance survival rates of adventitious roots of *Panax ginseng*. Hort. Environ. Biotech..

[72] Lee Y.Y., Balaraju K., Song J.Y., Yi J.Y., Lee S.Y., Lee J.R., Yoon M., Kim H.H. (2019). Cryopreservation of in vitro grown shoot tips of strawberry (*Fragaria × ananassa* Duch.) genetic resources by droplet-vitrification. Korean J. Plant Res..

[73] Yang X., Popova E., Shukla M.R., Saxena P. (2019). Root cryopreservation to biobank medicinal plants: A case study for *Hypericum perforatum* L. Vitr. Cell. Dev. Biol.-Plant.

[74] Lee H.-E., Popova E., Park H.-N., Park S.-U., Kim H.-H. (2021). Optimization of a cryopreservation method for the endangered Korean species *Pogostemon yatabeanus* using a systematic approach: The key role of ammonium and growth regulators. Plants.

[75] Popova E.V., Shukla M.R., McIntosh T., Saxena P.K. (2021). In vitro and cryobiotechnology approaches to safeguard *Lupinus rivularis* Douglas ex Lindl., an endangered plant in Canada. Agronomy.

[76] Kim H.H., Lee S.C. (2012). ‘Personalisation’ of droplet-vitrification protocols for plant cells: A systematic approach to optimizing chemical and osmotic effects. CryoLetters.

[77] Fahy G.M. (1977). Correlations between Cryoinjury in Mammalian Systems and Changes in the Composition and Properties of the Extracellular Milieu during Freezing. Ph.D. Thesis.

[78] Lee H., Kim H. (2022). Vigorous growing of donor plantlets by liquid overlay in subcultures is the key to cryopreservation of endangered species *Pogostemon yatabeanus*. Plants.

[79] Vandenbussche B., Weyens G., De Proft M. (2000). Cryopreservation of in vitro sugar beet (*Beta vulgaris* L.) shoot tips by a vitrification technique. Plant Cell Rep..

[80] Engelmann F. (2014). Cryopreservation of clonal crops: A review of key parameters. Acta Hortic..

[81] Dereuddre J., Fabre J., Bassaglia C. (1988). Resistance to freezing in liquid nitrogen of carnation (*Dianthus caryophyllus* L. var Eolo) apical and axillary shoot tips excised from different aged in vitro plantlets. Plant Cell Rep..

[82] Baek H.J., Kim H.H., Cho E.G., Chae Y.A., Engelmann F. (2003). Importance of explant size and origin and of preconditioning treatments for cryopreservation of garlic shoot apices by vitrification. CryoLetters.

[83] Yoon J.-W., Kim H.-H., Ko H.-C., Hwang H.-S., Hong E.-S., Cho E.-G., Engelmann F. (2006). Cryopreservation of cultivated and wild potato varieties by droplet vitrification: Effect of subculture of mother-plants and of preculture of shoot tips. CryoLetters.

[84] Bettoni J.C., Bonnart R., Shepherd A., Kretzschmar A.A., Volk G.M. (2019). Modifications to a *Vitis* shoot tip cryopreservation procedure: Effect of shoot tip size and use of cryoplates. CryoLetters.

[85] Guerra P.A., Souza E.H., Max D.A.S., Rossi M.L., Villalobos-Olivera A., Ledo C.A.S., Martinez-Montero M.E., Souza F.V.D. (2021). Morphoanatomical aspects of the starting material for the improvement of pineapple cryopreservation by the droplet-vitrification technique. An. Acad. Bras. Cienc..

[86] Pathirana R., Mathew L., McLachlan A. (2021). A simplified method for high recovery of kiwifruit (*Actinidia* spp.) shoot tips after droplet vitrification cryopreservation suitable for long-term conservation. Plant Cell Tiss. Organ. Cult..

[87] Reed B. (2018). Culture conditions are as important as the protocol in successful cryopreservation. Cryobiology.

[88] Engelmann F., Engelmann F., Takagi H. (2000). Importance of cryopreservation for the conservation of plant genetic resources. Cryopreservation of Tropical Germplasm. Current Research Progress and Application.

[89] Viseur J. (1987). Micropropagation of pear, *Pyrus communis* L. in a double-phase culture medum. Acta Hortic..

[90] Fujiwara K., Kira S., Kozai T. (1995). Contribution of photosynthesis to dry weight increase of in vitro potato cultures under different CO_2_ concentrations. Acta Hortic..

[91] Pullman G.S., Skryabina A. (2007). Liquid medium and liquid overlays improve embryogenic tissue initiation in conifers. Plant Cell Rep..

[92] Hussien F.A., Osman M.A., Idris T.I.M. (2014). The influence of liquid media support, gelling agents and liquid overlays on performance of in vitro cultures of ginger (*Zingiber officinale*). Intl. J. Sci. Res. Pub..

[93] Siddiqui Z.H., Mujib A., Maqsood M. (2011). Liquid overlaying improves somatic embryogenesis in *Catharanthus roseus*. Plant Cell Tiss. Organ Cult..

[94] Barraco G., Chatelet P., Balsemin E., Decourcelle T., Sylvestre I., Engelmann F. (2012). Cryopreservation of *Prunus cerasus* through vitrification and replacement of cold hardening with preculture on medium enriched with sucrose and/or glycerol. Sci. Hortic..

[95] Jitsuyama Y., Suzuki T., Harada T., Fujikawa S. (2002). Sucrose incubation increases freezing tolerance of asparagus (*Asparagus officinalis* L.) embryogenic cell suspensions. CryoLetters.

[96] Zhu G.Y., Geuns J.M.C., Dussert S., Swennen R., Panis B. (2006). Change in sugar, sterol and fatty acid composition in banana meristems caused by sucrose-induced acclimation and its effects on cryopreservation. Physiol. Plantarum.

[97] Carpentier S.C., Witters E., Laukens K., Van Onckelen H., Swennen R., Panis B. (2007). Banana (*Musa* spp.) as a model to study the meristem proteome: Acclimation to osmotic stress. Proteomics.

[98] Kim H.H., Cho E.G., Baek H.J., Kim C.Y., Keller E.R.J., Engelmann F. (2004). Cryopreservation of garlic meristems by vitrification: Effect of dehydration, unloading, rewarming and regrowth conditions. CryoLetters.

[99] Johnston J.W., Harding K., Benson E.E. (2007). Antioxidant status and genotypic tolerance of *Ribes* in vitro cultures to cryopreservation. Plant Sci..

[100] Ren L., Wang M.R., Wang Q.C. (2021). ROS-induced oxidative stress in plant cryopreservation: Occurrence and alleviation. Planta.

[101] Volk G.M., Henk A., Basu C. (2011). Gene expression in response to cryoprotectant and liquid nitrogen exposure in *Arabidopsis* shoot tips. Acta Hortic..

[102] Gross B.L., Henk A.D., Bonnart R., Volk G.M. (2017). Changes in transcript expression patterns as a result of cryoprotectant treatment and liquid nitrogen exposure in *Arabidopsis* shoot tips. Plant Cell Rep..

[103] Thomson L.K., Fleming S.D., Aiken R.J., De Iuliis G.N., Zieschang J.A., Clark A.M. (2009). Cryopreservation-induced human sperm DNA damage is predominantly mediated by oxidative stress rather than apoptosis. Hum. Reprod..

[104] Funnekotter B., Colville L., Kaczmarczyk A., Turner S.R., Bunn E., Mancera R.L. (2017). Monitoring of oxidative status in three native Australian species during cold acclimation and cryopreservation. Plant Cell Rep..

[105] Lee H., Choi B., Oh S., Park H., Popova E., Paik M.-J., Kim H. (2023). Dynamics of Organic Acids during the Droplet-Vitrification Cryopreservation Procedure Can Be a Signature of Oxidative Stress in *Pogostemon yatabeanus*. Plants.

[106] Kuriyama A., Watanabe K., Kawata K., Kawai F., Kanamori M. (1996). Sensitivity of cryopreserved *Lavandula vera* cells to ammonium ion. J. Plant Physiol..

[107] Reed B. (2014). Antioxidants and cryopreservation, the new normal?. Acta Hortic..

[108] Whelehan L.M., Dalziell E.L., Bunn E., Mancera R.L., Funnekotter B. (2022). How does metabolic rate in plant shoot tips change after cryopreservation?. Cryobiology.

[109] Yang S., Hao D., Jin M., Li Y., Liu Z., Huang Y., Chen T., Su Y. (2020). Internal ammonium excess induces ROS-mediated reactions and causes carbon scarcity in rice. BMC Plant Biol..

[110] Pennycooke J.C., Towill L.E. (2001). Medium alterations improve regrowth of sweet potato (*Ipomea batatas* L. Lam.) shoot cryopreserved by vitrification and encapsulation-dehydration. CryoLetters.

[111] Martinez-Montero M.E., Harding K., Barh D., Khan M., Davies E. (2015). Cryobionomics: Evaluating the Concept in Plant Cryopreservation. PlantOmics: The Omics of Plant Science.

